# Bacterial extracellular vesicles repress the vascular protective factor RNase1 in human lung endothelial cells

**DOI:** 10.1186/s12964-023-01131-2

**Published:** 2023-05-15

**Authors:** Katrin Laakmann, Jorina Mona Eckersberg, Moritz Hapke, Marie Wiegand, Jeff Bierwagen, Isabell Beinborn, Christian Preußer, Elke Pogge von Strandmann, Thomas Heimerl, Bernd Schmeck, Anna Lena Jung

**Affiliations:** 1 Institute for Lung Research, Universities of Giessen and Marburg Lung Center, Philipps-University Marburg, German Center for Lung Research (DZL), Marburg, Germany; 2 Institute for Tumor Immunology and Core Facility – Extracellular Vesicles, Philipps-University Marburg, Marburg, Germany; 3 Center for Synthetic Microbiology (SYNMIKRO), Philipps-University Marburg, Marburg, Germany; 4 Core Facility Flow Cytometry – Bacterial Vesicles, Philipps-University Marburg, Marburg, Germany; 5 Department of Pulmonary and Critical Care Medicine, Philipps-University Marburg, Marburg, Germany; 6 Member of the German Center for Infectious Disease Research (DZIF), Marburg, Germany

**Keywords:** Sepsis, OMV, Ribonuclease 1, Endothelium, Inflammation, TLR4, Polymyxin B, p38

## Abstract

**Background:**

Sepsis is one of the leading causes of death worldwide and characterized by blood stream infections associated with a dysregulated host response and endothelial cell (EC) dysfunction. Ribonuclease 1 (RNase1) acts as a protective factor of vascular homeostasis and is known to be repressed by massive and persistent inflammation, associated to the development of vascular pathologies. Bacterial extracellular vesicles (bEVs) are released upon infection and may interact with ECs to mediate EC barrier dysfunction. Here, we investigated the impact of bEVs of sepsis-related pathogens on human EC RNase1 regulation.

**Methods:**

bEVs from sepsis-associated bacteria were isolated via ultrafiltration and size exclusion chromatography and used for stimulation of human lung microvascular ECs combined with and without signaling pathway inhibitor treatments.

**Results:**

bEVs from *Escherichia coli*, *Klebsiella pneumoniae* and *Salmonella enterica* serovar Typhimurium significantly reduced RNase1 mRNA and protein expression and activated ECs, while TLR2-inducing bEVs from *Streptococcus pneumoniae* did not. These effects were mediated via LPS-dependent TLR4 signaling cascades as they could be blocked by Polymyxin B. Additionally, LPS-free *ClearColi*™ had no impact on RNase1. Further characterization of TLR4 downstream pathways involving NF-кB and p38, as well as JAK1/STAT1 signaling, revealed that RNase1 mRNA regulation is mediated via a p38-dependent mechanism.

**Conclusion:**

Blood stream bEVs from gram-negative, sepsis-associated bacteria reduce the vascular protective factor RNase1, opening new avenues for therapeutical intervention of EC dysfunction via promotion of RNase1 integrity.

Video Abstract

**Supplementary Information:**

The online version contains supplementary material available at 10.1186/s12964-023-01131-2.

## Introduction

Sepsis is one of the leading causes of death worldwide with approximately 50 million cases per year [1]. Development and progression of sepsis is mainly caused by bacterial infections of the lung, urinary tract, skin and intestine [1–5]. Accordingly, frequent pathogens like gram-positive *Streptococcus pneumoniae* (*Sp*) as well as gram-negative, increasingly antibiotic-resistant *Enterobacteriaceae* like *Escherichia coli* (*Ec*), *Klebsiella pneumoniae* (*Kp*) or *Salmonella enterica* (*Sal*) are of major interest [6, 7]. Exemplarily, severe lung infections cause a breakdown of the alveolar membrane and enable the invasion of bacterial pathogens into the bloodstream [3, 7]. These systemic infections provoke an imbalanced immune response of the host that finally ends up in vascular barrier breakdown, multi-organ failure and death [8, 9]. In this regard, sepsis progression is characterized by excessive production of pro-inflammatory cytokines and a pro-coagulant state of the vasculature that further promotes endothelial cell (EC) dysfunction [2, 3, 5].

Besides classical inflammatory factors like cytokines or chemokines, extracellular vesicles (EVs) play an essential role during infection and inflammation as novel mediators of intercellular communication [10]. EVs are small, nano- to micrometer sized, spherical membrane enclosed structures that can be released by eukaryotic host cells as well as bacteria (bEVs; [11]). bEVs from bacteria can further be separated into two classes, outer membrane vesicles (OMVs) that are secreted by gram-negative bacteria and membrane vesicles (MVs) that are secreted by gram-positive bacteria [11–13]. In respect to pneumonia, various studies describe a wide range of bEV functions in host–pathogen interactions (reviewed in: [11]) and an additional impact of bEVs in blood stream infection is currently under consideration. Thereby, bEVs can either be released by circulating pathogens, but are also capable of reaching distant organs independently of the secreting bacteria [14], where they fulfill an essential role in progression of sepsis-associated dysfunction of the endothelium [15–20]. The pathogen-EC interaction is primarily regulated by bacterial immune agonists like lipopolysaccharide (LPS) or lipoprotein that are also associated to the bEV surface [21]. Those known pattern-recognition receptor ligands can further induce inflammation and intracellular signaling in the endothelium, such as activation of Toll-like receptors (TLRs) that promote disruption of the EC barrier and immune function [16, 22]. Cell membrane-bound TLRs like TLR2 or TLR4 can sense bEVs and induce downstream signaling via the MyD88/IRAK-1 axis that is mediated by IRAK-1 degradation to further promote NF-κB translocation to the nucleus as well as p38 phosphorylation and translocation to activate associated gene expression. Additionally, TLR4 can also induce TRIF-dependent signaling upon endocytosis that promotes IRF3/7-mediated type I interferon (IFN) production and subsequent activation o the f type I IFN receptor (IFNAR) and the JAK/STAT pathway (Fig. S1) [23–25].

ECs function as a protective barrier to separate the blood from the surrounding tissue and act as regulators of vascular homeostasis [26–28]. Upon inflammation, ECs get activated, which is characterized for instance by secretion of pro- and antithrombotic factors as well as upregulation of pro-inflammatory mediators like adhesion molecules and cytokines (e.g., intercellular adhesion molecule 1 (ICAM-1), C-X-C motif chemokine ligand 8 (CXCL8), C-X-C motif chemokine ligand 10 (CXCL10)) [15, 26–29]. Thereby, massive and persistent inflammation can tremendously harm the homeostatic function of the endothelium and is involved in development of vascular dysfunctions such as sepsis-associated formation of micro-thrombosis or intravascular coagulation [15, 27].

Endothelial Ribonuclease 1 (RNase1) is known as an important vessel- and tissue-protective factor [30–33], countering the damage associated molecular pattern extracellular RNA (eRNA) to prevent excessive EC inflammation [34]. However, massive inflammation results in eRNA-induced EC inflammation and RNase1 repression, effected by massive amounts of pro-inflammatory mediators like tumor necrosis factor alpha (TNF-α) [34–36], that promotes EC dysfunction and development of vascular pathologies including thrombosis [30, 34, 37–41]. Besides these data, recent studies also investigated the role of the RNase1-eRNA system in sepsis suggesting a potential RNase1 repression during disease progression as well as a protective function of RNase1 during sepsis-associated tissue- and organ damage [42, 43]. Altogether, the current literature suggests a causal interaction between sepsis-associated EC dysfunction and RNase1 repression that is still insufficiently studied.

Here, we investigated the impact of bEVs from different sepsis-associated bacterial pathogens on the regulation of the vessel-protective factor RNase1 and the underlying signaling cascades in human lung ECs. In this study, we found that OMVs from the gram-negative bacteria *Escherichia coli*, *Klebsiella pneumoniae* and *Salmonella enterica* serovar Typhimurium repress endothelial RNase1 via an LPS-induced TLR4-IRAK-1 and p38-mediated mechanism to promote EC inflammation that favors development of endothelial dysfunction independent of the investigated donor bacteria.

## Material and methods

### Bacterial vesicle generation and isolation

*Escherichia coli* (#25922, *Ec*), *Klebsiella pneumoniae* (#700721/MGH78578, *Kp*), and *Salmonella* *enterica* serovar Typhimurium (#14028, *Sal*) were purchased from American Type Culture Collection (ATCC; Manassas, USA). *ClearColi*™ BL21 (*cC*) were obtained from BioCat GmbH (Heidelberg, Germany). *Streptococcus pneumoniae* (D39, *Sp*) were kindly provided by Sven Hammerschmidt (University Greifswald, Germany). *Ec*, *Kp* and *Sal* were cultivated on MacConkey agar plates, *cC* in LB with 1% NaCl and *Sp* on blood agar plates overnight at 37°C and 5% CO_2_. Bacteria were then used for inoculation of liquid culture media (LB: *Ec*, *Kp, Sal*; LB + 1% NaCl: *cC*; THY: *Sp*) and grown until they reached the late exponential phase with shaking at 160 rpm and 37°C (MaxQ 6000, Thermo Fisher Scientific, Waltham, USA) excluding *Sp*, which were cultivated without shaking. To obtain a medium control for following stimulation experiments, LB medium and THY medium was handled in parallel to bacterial liquid cultures during vesicle preparation serving as medium controls without bacterial growth. Next, bacterial cultures, LB and THY media were centrifuged three times at 4,800 xg for 20 min at 4 °C (Multifuge X3R, Thermo Fisher Scientific) and residual bacteria were removed from the supernatant by using a 0.22 µm pore sterile filter unit. Bacterial vesicles and medium were further processed by ultrafiltration and size exclusion chromatography (UF-SEC) as follows: Bacterial/medium supernatants were concentrated using a 100 kDa molecular weight cut-off filter (Merck Millipore, Burlington, Sigma Aldrich, St. Lois, USA) to a final volume of 500 µl, which was further processed by size exclusion chromatography using the qEVoriginal/ 70 nm Gen 2 columns (IZON Science LTD, Lyon, France). Vesicle elution was proceeded using 0.1 µm filtered PBS and 24 SEC-fractions were collected (500 µl/fraction). Vesicle enriched fractions (7–12) were concentrated to a final volume between 200 and 400 µl using molecular weight cut-off filters (Merck Millipore) and particle concentration was determined by nano-flow cytometry (nanoFCM; NanoFCM Co., Ltd, Nottingham, UK). Equal amounts of vesicles per cell (multiplicity of vesicles of 1000; MOV_1000_) were used for stimulation experiments. Each bacterial vesicle preparation was checked for contaminating bacteria by plating on blood agar plates. Vesicles were aliquoted and stored at -20°C as working stocks.

### nanoFCM

nanoFCM measurements of bEV preparations to determine bEV concentration and size distribution were performed as previously described by Bierwagen et al. 2023 [44].

### Transmission Electron Microscopy (TEM)

TEM was performed to validate intact vesicle structures as previously described by Bierwagen *et al.* 2023 [44].

### Cell culture

Cells used in this study were cultivated in a humidified incubator at 37°C with 5% CO_2_. Human microvascular lung endothelial cells (HULEC-5a) (CRL-3244™, ATCC) were cultured in human microvascular endothelial cell medium MCDB 131 (Gibco™, Thermo Fisher Scientific) supplemented with 10% fetal calf serum (Capricorn Scientific GmbH, Ebsdorfergrund, Germany), 1% penicillin and streptomycin (Gibco™, Thermo Fisher Scientific), 10 mM GlutaMax™ (Gibco®, Thermo Fisher Scientific), 10 ng/ml EGF (Merck Millipore, Sigma Aldrich) and 1 µg/ml hydrocortisone (Th. Geyer Ingredients GmbH & Co. KG, Höxter, Germany). Cells were cultured up to passage 20 for all experiments.

### Stimulation of endothelial cells

Cells were seeded with 3.8*10^4^ cells/cm^2^ overnight followed by stimulation for 16 or 24 h (mRNA expression and ELISA) or 0–180 min (Western Blot) as indicated: TNF-α (10 ng/ml) (R&D Systems, Inc., Minneapolis, USA), LPS from *Escherichia coli* O111:B4 (100 ng/ml) (cell culture grade, Sigma Aldrich), LPS from *Salmonella minnesota* R595 (100 ng/ml) (cell culture grade, Enzo Life Sciences, Lausen, Switzerland), IFN-ɣ (250 ng/ml) (Promo Cell, Heidelberg, Germany) or with vesicles from gram-negative bacteria (OMVs) from *Escherichia coli* (*Ec*OMV), *Klebsiella pneumoniae* (*Kp*OMV), *Salmonella enterica* serovar Typhimurium (*Sal*OMV), *Clear coli* (*cC*OMV) or MVs from gram-positive *Streptococcus pneumoniae* (*Sp*MV) with MOV_1000_, respectively. For Polymyxin B treatment (PB; Merck Millipore), OMVs were preincubated for 1 h with the LPS neutralizing antibiotic PB (20 µg/ml) (Merck Millipore) followed by stimulation as indicated. For inhibitor experiments, HULEC-5a were pretreated for 1 h with the JAK1 inhibitor Ruxolitinib (5 µM; JAKi) (Biozol Diagnostics Vertrieb GmbH, Eching, Germany), the NF-κB inhibitor BAY11-7082 (5 µM; NF-κBi) or the p38 inhibitor SB202190 (10 µM; p38i) (Merck Millipore) prior to indicated stimulation. Dimethyl sulfoxide (DMSO, Carl Roth GmbH + Co. KG, Karlsruhe, Germany) served as solvent control.

### RNA isolation and quantitative reverse transcription PCR

Total RNA was isolated from HULEC-5a and cDNA was generated as described previously [36]. mRNA transcript expression of RNase1, CXCL8, ICAM-1, CXCL10, TLR2 and TLR4 was analyzed by quantitative reverse transcription PCR (qPCR) compared to RPS18 that served as internal control. Respective primer pairs are listed in Table 1 (Metabion international AG, Planegg/Steinkirchen, Germany). qPCR was performed using LUNA^®^ Universal qPCR Master Mix (New England Biolabs, Ipswich, USA) and the QuantStudio^TM^ System and QuantStudio^TM^ Design & Analysis Software v1.3.1 (both Thermo Fisher Scientific) according to the manufacturer’s instructions. The 2^-ΔΔct^ method was used for calculation of the fold-induction and qPCR results were normalized to the corresponding control cells [45].

**Table 1:** Primer sequences 5′➔3’

Primer	Forward	Reverse
CXCL8	ACTGAGAGTGATTGAGAGTGGAC	AACCCTCTGCACCCAGTTTTC
CXCL10	CTGCCATTCTGATTTGCTGCC	GATGCAGGTACAGCGTACAG
ICAM-1	CTCAGTCAGTGTGACCGC	CCTTCTGAGACCTCTGGC
RNase1	GCTGCAGATCCAGGCTTTTCTGGG	AATTTCTTGGCCCGGGATTC
RPS18	GCGGCGGAAAATAGCCTTTG	GATCACACGTTCCACCTCATC
TLR2	GGCCAGCAAATTACCTGTGTG	AGGCGGACATCCTGAACCT
TLR4	TGAGACCAGAAAGCTGGGAG	ACTCTGGATGGGGTTTCCTG

### LDH-assay

Cytotoxicity of HULEC-5a upon stimulation was determined via lactate dehydrogenase (LDH) release into supernatants compared to a total lysis (TL) representing 100% cell death using the Pierce™ LDH Cytotoxicity Assay Kit (Roche, Mannheim, Germany) according to the manufacturer’s protocol. The absorbance was measured using the infinite F200Pro microplate reader (Tecan, Männedorf, Switzerland).

### Protein isolation for Western Blot

HULEC-5a were seeded and stimulated as described before for 0–180 min as indicated. For isolation of total protein, cells were washed once with PBS and stored dry at -20°C until further processing. Cells were scraped and lysed in RIPA buffer (containing cOmplete™ mini protease inhibitor cocktail and PhosSTOP™, Merck Millipore) followed by sonication via ultrasound for 5 min (30 s on/off at 4°C). Samples were centrifuged for 20 min at 13,000 xg at 4 °C to remove cellular debris. For fractionation of cytosolic and nuclear proteins, HULEC-5a were seeded and stimulated as described above for 30 min. After stimulation, cells were scraped in 1 ml PBS and centrifuged at 240 xg at 4°C for 2 min to pellet cells. The supernatant was discarded, and the pellet was resuspended in fractionation buffer A (10 mM HEPES, pH 7.9, 10 mM KCl, 0.1 mM EDTA, 0.1 mM EGTA, supplemented with protease inhibitor as well as 0.5 mM DTT) and incubated for 15 min on ice. Cell disruption was additionally promoted by multiple aspirating of the suspension using a 26G needle and syringe, followed by centrifugation for 2 min at 4,400 xg at 4°C. For cytosolic protein isolation, the supernatant was centrifuged for additional 20 min at 20,000 xg at 4°C and stored at -80°C until further use. For nuclear protein isolation, the pellet was washed twice with fractionation buffer A and the nuclei containing pellet was resuspended in fractionation buffer B (20 mM HEPES, pH 7.9, 400 mM NaCl, 1 mM EDTA, 1 mM EGTA) and incubated for 30–60 min at 4 °C with shaking at 1,000 rpm. Samples were centrifuged for 20 min at 20,000 xg at 4°C and supernatants were stored at -80°C. Protein concentration of all samples was measured using the Pierce BCA protein assay kit according to the manufacturer’s instructions (Thermo Fisher Scientific). Twenty-five microgram protein per sample and fifteen microgram for fractionation samples were loaded and separated on a 10% or 12.5% SDS PAGE gel (30 min at 80 V and subsequently ~ 120 min for total protein samples or ~ 180 min for fractionation samples at 120 V), respectively, followed by protein transfer and immobilization via wet blot procedure using a PerfectBlue™ Tank Electro Blotter (VWR International, Radnor, USA) and Towbin buffer on 0.2 µm nitrocellulose membrane (GE Healthcare, Chicago, USA) for 1 h with 100 V at 4°C. Membranes were blocked and exposed to antibodies targeting IRAK-1 (4359S), phospho-p38 (Thr180/Tyr182) (9211S), p38 (9212S), STAT1 (D1K9Y, 14994S), phospho-STAT1 (Tyr701) (58D6, 9167S) (Cell Signaling, Cambridge, UK), p65 (F-6; sc-8008), Lamin A/C (H-110; sc-20681), α1c-Tubulin (MH-87, sc-134239) and β-actin (I-19, sc-1616) (Santa Cruz Biotechnology, Heidelberg, Germany) followed by incubation with respective secondary, HRP-conjugated antibodies: mouse anti-rabbit IgG-HRP (L27A9, 5127S; Cell Signaling) or anti-mouse m-IgGк BP-HRP (sc-516102; Santa Cruz Biotechnology). Chemiluminescence was detected using Amersham™ ECL™ Prime Western Blotting Detection Reagent (RPN2236, cytiva, Merck Millipore) and visualized by the ADVANCED Fluorescence and ECL Imager (Intas Science Imaging Instruments, Germany).

### ELISA

Supernatants of stimulated HULEC-5a were used for protein detection of CXCL8 and RNase1 via ELISA. CXCL8 ELISA was performed as recommended by the manufacturer’s protocol using the BD OptEIA^TM^ Human IL-8 ELISA Set (555244, BD Biosciences, Franklin Lakes, USA). RNase1 ELISA was performed using the human Ribonuclease A Matched ELISA Antibody Pair Set (SEK13468, Sino Biological, Inc., Beijing, China) as recommended by the manufacturer’s instructions. Detection of HRP-mediated signal was performed using BD OptEIA^TM^ TMB Substrate Reagent Set (555214, BD Biosciences) and the absorbance was measured using the microplate reader infinite F200Pro (Tecan).

### Statistical analyses

Statistical analyses were performed using GraphPad Prism Version 9.5.0 (730) (GraphPad Software, La Jolla, CA, USA). qPCR results are expressed as log2 transformed data with line at mean. ELISA results are expressed as linear data, line at mean. One-way or two-way ANOVA were performed as indicated with subsequent multiple comparison using recommended post-tests as indicated in the figure legend. Results were considered significant at *p* ≤ 0.05, which is labelled with * or # in the figures.

### Availability of data and materials

All data generated or analyzed during this study are included in this article and its supplementary file.

## Results

### Characterization of bacterial vesicles

**Fig. 1:**
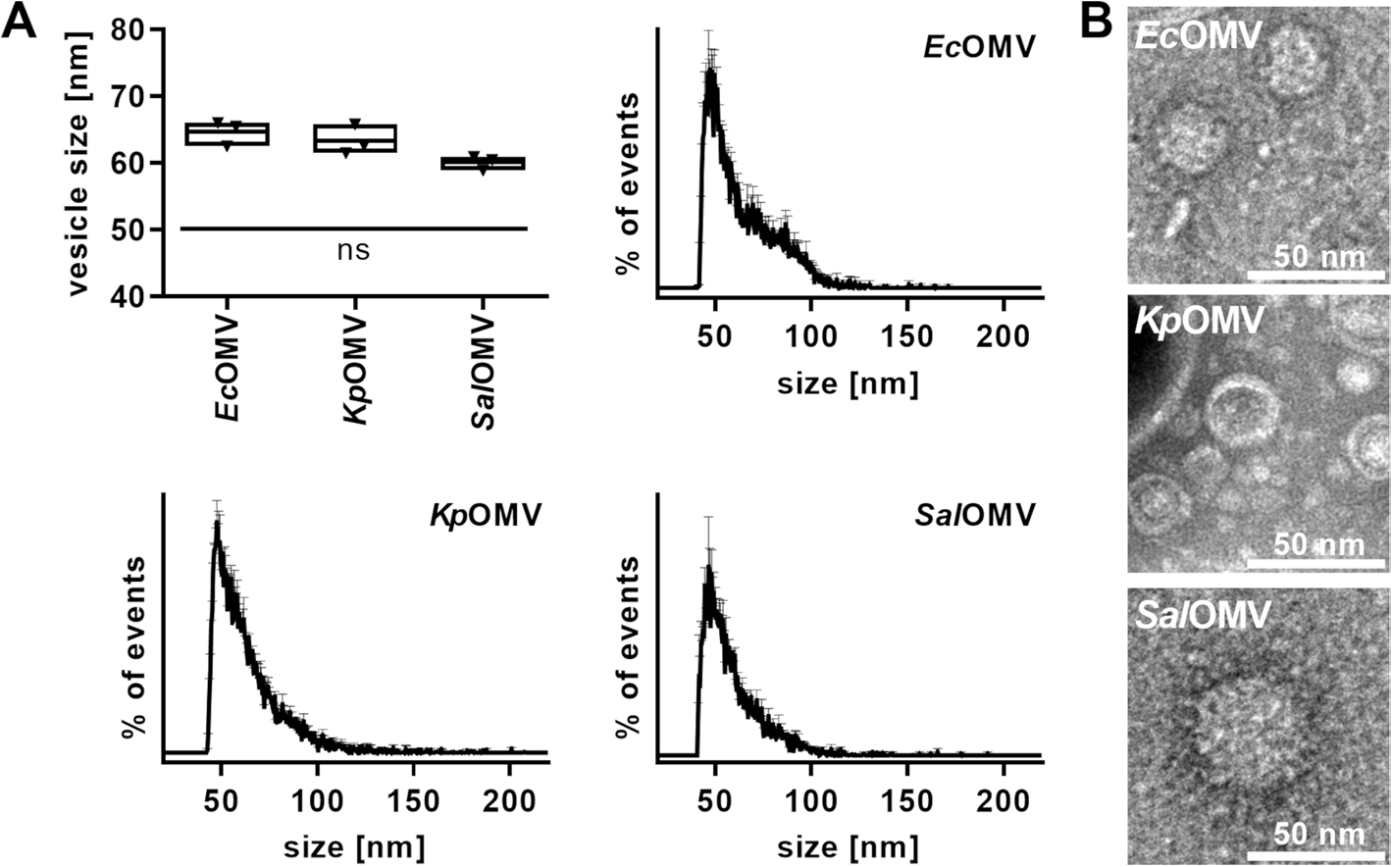
Characterization of bacterial outer membrane vesicles. **A)** Mean size of SEC-purified OMVs from *Escherichia coli* (*Ec*OMV), *Klebsiella pneumoniae (Kp*OMVs) and *Salmonella enterica* serovar Typhimurium (*Sal*OMV) and their size distribution profile was determined by nanoFCM. *n* = 3, line at mean, One-way ANOVA with Tukey‘s multiple comparison test; ns: not significant. **B)** Vesicle shape and structure was validated by transmission electron microscopy (TEM). Scale bar: 50 nm

To investigate the impact of bacterial vesicles from the sepsis-associated gram-negative pathogens *Escherichia coli* (*Ec*OMVs), *Klebsiella pneumoniae* (*Kp*OMVs) and *Salmonella* *enterica* serovar Typhimurium (*Sal*OMVs) as well as the gram-positive pathogen *Streptococcus pneumoniae* (*Sp*MVs) on endothelial RNase1, OMVs and MVs were isolated from liquid bacterial culture via UF-SEC (Fig. S2A). Particle concentration and size distribution of isolated vesicles was investigated by nanoFCM. No significant differences were observed between the different vesicle types showing mean sizes of approximately 60–70 nm as well as their size distribution profile, peaking at ~ 50 nm (Fig. 1A and Fig. S2B-D). Further characterization of isolated bacterial vesicles was performed by TEM imaging showing intact, spherical, membrane enclosed structures for all tested vesicle types (Fig. 1B).

### OMVs from *Ec*, *Kp*, *Sal* repress RNase1 and activate the endothelium

**Fig. 2:**
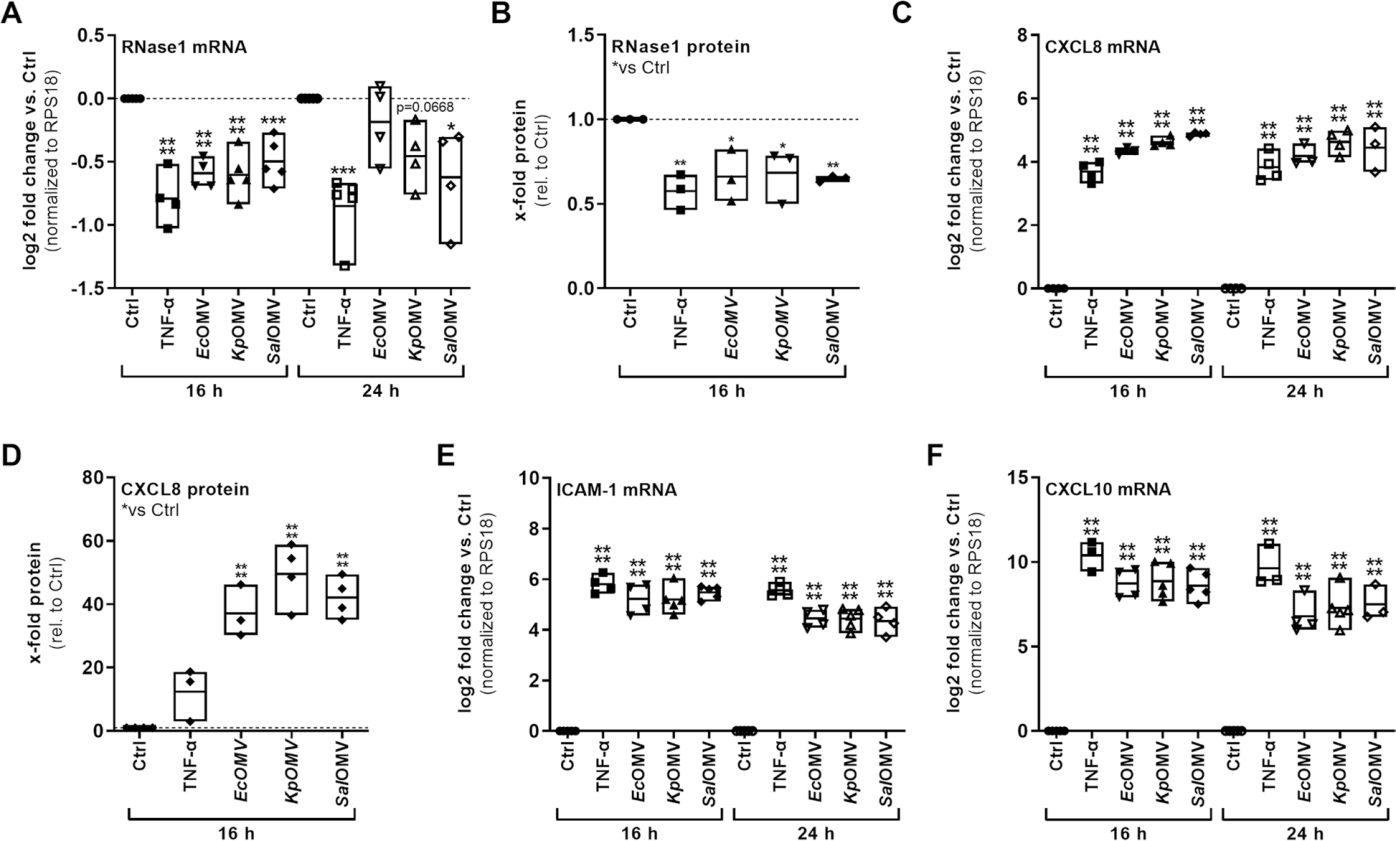
OMVs repress endothelial RNase1 and induce proinflammatory EC activation. Human lung microvascular ECs (HULEC-5a) were stimulated for 16 h and 24 h with SEC-purified OMVs (MOV_1000_) from *Ec*, *Kp* and *Sal* or TNF-α (10 ng/ml) or left untreated as control (Ctrl). mRNA expression of **A**) RNase1 **C**) CXCL8, **E**) ICAM-1 and **F**) CXCL10 was determined by qPCR, normalized to RPS18 and untreated cells (Ctrl). **B)** RNase1 and **D)** CXCL8 protein release in supernatants of 16 h stimulated HULEC-5a was measured by ELISA, depicted as x-fold protein relative to control (Ctrl). *n* = 3–4, line at mean, One-way ANOVA with Dunnett‘s multiple comparison test compared to respective Ctrl. *p < 0.05, **p < 0.01, ***p < 0.001, ****p < 0.0001

To analyze the regulatory potential of OMVs and MVs on endothelial RNase1 and their impact on proinflammatory EC activation, HULEC-5a were exposed to TNF-α (10 ng/ml) as positive control [36], *Ec*OMVs, *Kp*OMVs, *Sal*OMVs and *Sp*MVs with MOV_1000_ or left untreated as control (Ctrl) for 16 and 24 h (Fig. 2, Figs. S3A and S4). UF-SEC processed LB- or THY-medium served as medium control to assess potential effects of medium components on EC activation (Fig. S3B-C). Interestingly, RNase1 mRNA expression was significantly repressed by *Ec*OMVs, *Kp*OMVs, *Sal*OMVs treatment after 16 h, similar to stimulation with the RNase1-repressive cytokine TNF-α. This effect was less prominent at 24 h, although RNase1 mRNA remained suppressed (Fig. 2A). In contrast, no effect on RNase1 mRNA could be observed by stimulation with the gram-positive *Sp*MVs (Fig. S4A) as well as the LB and THY-medium controls (Fig. S3C). Similar to TNF-α, OMVs from *Ec*, *Kp*, *Sal* also repressed secretion of RNase1 protein measured by ELISA after 16 h stimulation (Fig. 2B), while *Sp*MV did not regulate RNase1 protein levels (Fig. S4B). Besides RNase1 regulation, all OMVs proinflammatory activated HULEC-5a after 16 and 24 h stimulation, as indicated by mRNA expression of CXCL8, ICAM-1 and CXCL10 (Fig. 2C, E–F), while this was not the case for *Sp*MV (Fig. S4C-E). Additionally, protein secretion of CXCL8 was also increased by OMV treatment (Fig. 2D). To ensure cell viability upon OMV and MV treatment, cytotoxicity of respective stimulants was obtained by LDH assay showing no significant increase in cytotoxicity upon exposure above a threshold of 30% cytotoxicity (Fig. S3A-B). Accordingly, OMVs from sepsis-associated gram-negative bacteria *Ec*, *Kp* and *Sal* specifically repressed endothelial RNase1 and activated human lung ECs, in contrast to gram-positive MVs from *Sp*. Based on these findings, further analysis focused on 16 h OMV treatment due to the observed significant RNase1 regulation in conjunction with a strong proinflammatory activation of the cells.

### OMVs induce a TLR4-dependent signaling cascade in human ECs to repress RNase1

To understand the underlying mechanisms, we investigated the basal mRNA expression of specific TLRs that are needed to sense those bEV types on the cell surface: TLR2 for *Sp*MVs and TLR4 for all tested OMV types. Basal mRNA expression of TLR2 and TLR4 was investigated by qPCR. These data revealed substantial differences in TLR2 and TLR4 mRNA expression in HULEC-5a with low abundance for TLR2 compared to high abundance for TLR4 (Fig. S5). Thus, unresponsiveness of HULEC-5a to *Sp*MV might be associated to the low availability of TLR2, while abundant TLR4 expression is a prerequisite for the responsiveness of these cells to *Ec*, *Kp* and *Sal* OMVs. To further unravel the intracellular signaling cascade that transmits OMV-mediated RNase1 repression, we addressed LPS that is exposed on the OMV surface as potent TLR4 agonist to induce proinflammatory signaling cascades via either the MyD88/IRAK-1 or the TRIF axis (Fig. S1) [23–25].

**Fig. 3:**
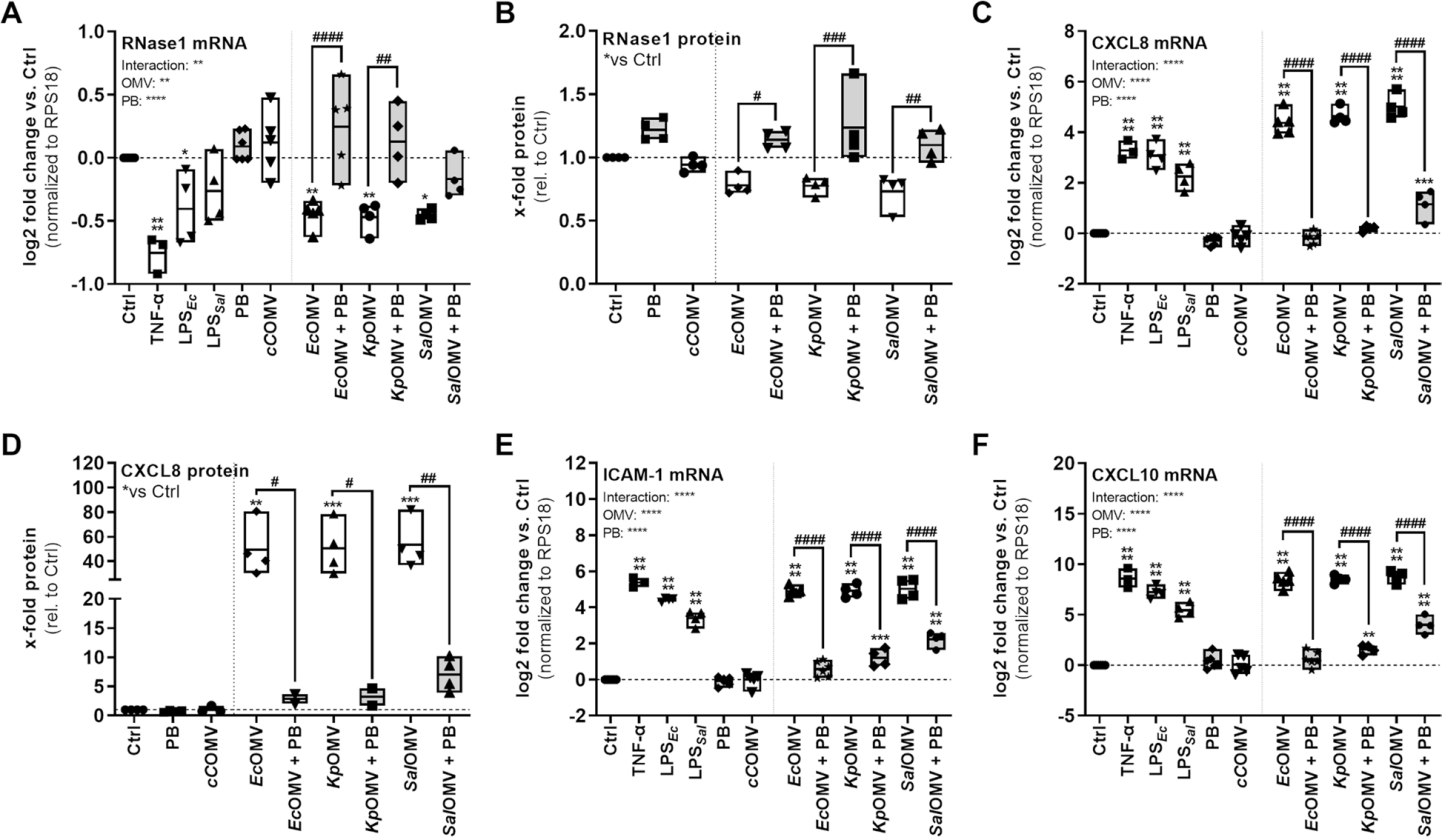
OMVs repress endothelial RNase1 and induce proinflammatory EC activation in a TLR4-dependent manner. SEC-purified OMVs from *Ec*, *Kp* and *Sal* were pretreated for 1 h with 20 µg/ml Polymyxin B (PB) and further used for stimulation of HULEC-5a (MOV_1000_) for 16 h, as well as TNF-α (10 ng/ml), LPS from *Ec* (LPS_*Ec*_) or *Sal* (LPS_*Sal*_) (100 ng/ml) or left untreated as control (Ctrl). mRNA expression of **A**) RNase1, **C**) CXCL8, **E**) ICAM-1 and **F**) CXCL10 was determined by qPCR, normalized to RPS18 and Ctrl. *n* = 3–5, line at mean, Two-way ANOVA with Tukey ‘s multiple comparison test. **B)** RNase1 protein release and **D**) CXCL8 protein release from supernatants of 16 h stimulated HULEC-5a was measured by ELISA, depicted as x-fold protein relative to Ctrl. *n* = 4, line at mean, One-way ANOVA with Tukey ‘s multiple comparison test. * compared to corresponding Ctrl, # as indicated. *p < 0.05, **p < 0.01, ***p < 0.001, ****p < 0.0001. #*p* < 0.05, ##*p* < 0.01, ####*p* < 0.0001

To block the potential LPS-mediated TLR4 induction by OMVs, the LPS neutralizing antibiotic Polymyxin B (PB) was used for further analysis (Fig. S1, Fig. 3) [46, 47]. *Ec*OMVs, *Kp*OMVs and *Sal*OMVs (MOV_1000_) were preincubated with PB (20 µg/ml) for 1 h, prior to 16 h stimulation of HULEC-5a with untreated or PB-pretreated OMVs. For comparison purposes, cells were stimulated with LPS from *E. coli* (LPS_*Ec*_) or *Salmonella* (LPS_*Sal*_) (100 ng/ml), TNF-α (10 ng/ml) or left unstimulated (Ctrl). As an additional control, OMVs from endotoxin free *Clear coli* (*cC*OMVs), that carry a modified lipid A and have therefore a non-functional version of LPS, served as control as these bacteria are not able to elicit TLR4-mediated endotoxic responses [48]. *cC*OMVs were comparable in size to all other used OMVs and did not show any difference in their size distribution profile (Fig. S2B and D).

In accordance with previous results, TNF-α and OMVs significantly reduced RNase1 mRNA expression, while LPS from *Ec* and *Sal* only showed tendencies of RNase1 repression. Compared to that, stimulation with *cC*OMVs and the LPS-blocking agent PB did not influence RNase1 mRNA expression. Remarkably, stimulation of HULEC-5a with PB-pretreated OMVs (grey bars) significantly recovered RNase1 mRNA compared to the respective untreated OMVs (white bars) for *Ec*, *Kp* and *Sal*OMVs (Fig. 3A). Similar results were observed on protein level, where TNF-α and untreated OMVs, except *cC*OMVs, repressed RNase1 protein release which could be recovered by OMV-pretreatment with PB (Fig. 3B). In addition to RNase1 regulation, TNF-α, LPS_*Ec*_, LPS_*Sal*_ and untreated OMVs, except *cC*OMVs, significantly induced proinflammatory EC activation as demonstrated by CXCL8 mRNA induction and protein release (Fig. 3C and D), ICAM-1 mRNA (Fig. 3E) and CXCL10 mRNA (Fig. 3F) compared to PB and Ctrl treatment. In addition, PB-pretreated OMVs were also not capable to induce a strong proinflammatory response. Additionally, none of the stimulations induced significant changes in cytotoxicity (Fig. S3D).

**Fig. 4:**
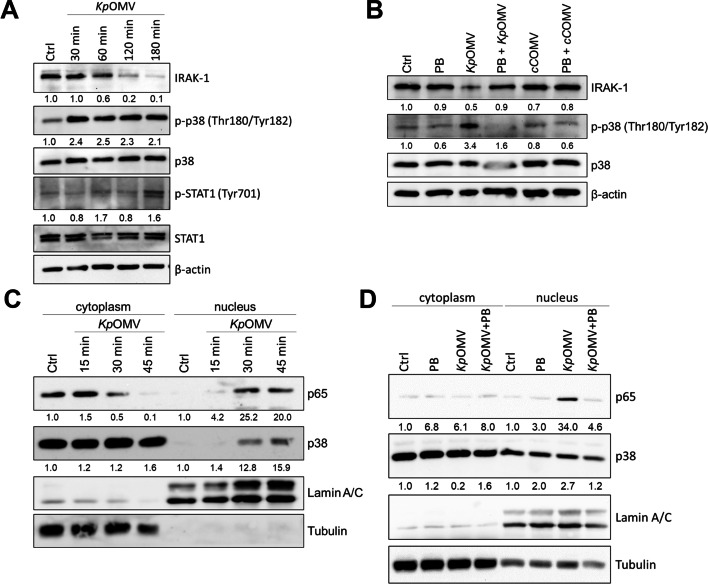
OMVs induce proinflammatory signaling cascades in human lung ECs. **A)** Human lung microvascular ECs (HULEC-5a) were stimulated with SEC-purified *Kp*OMVs (MOV_1000_) or left untreated as control (Ctrl). Expression and phosphorylation of IRAK-1, p38 and STAT1 after OMV exposure for up to 180 min were determined by Western Blot. β-actin served as a loading control. **B)** HULEC-5a were stimulated for 180 min with *Kp* or *cC*OMVs (MOV_1000_) alone or in combination with PB (20 µg/ml) or left untreated for Ctrl. Expression and phosphorylation of IRAK-1 and p38 was determined by Western Blot. β-actin served as a loading control. **C)** HULEC-5a were incubated with *Kp*OMV for up to 45 min or **D**) incubated with *Kp*OMV for 30 min with or without 1 h pretreatment with PB (20 µg/ml). Samples were fractionated into cytosolic and nuclear proteins and analyzed by Western Blot for p65 and p38. Tubulin served as a cytoplasmic loading control, while Lamin A/C served as a nuclear loading control. One representative result of three biological independent replicates is shown

To further validate the previous observations on protein level, activation of the TLR4-associated intracellular signaling molecules (Fig. S1) in *Kp*OMV-stimulated ECs (MOV_1000_ for 0–180 min) was analyzed. Degradation of IRAK-1, phosphorylation of p38 (at threonine 180 and tyrosine 182) and phosphorylation of STAT1 (at tyrosine 701) were investigated by Western Blot along with the loading control β-actin (Fig. 4A). IRAK-1 degradation started at approximately 60 min after OMV exposure, while phosphorylation of p38 already increased 30 min after OMV exposure. Phosphorylation of STAT1 was prominent after 180 min of stimulation. To investigate the involvement of TLR4 in the observed activation pattern in ECs, cells were further stimulated with *Kp*OMVs alone or in combination with PB (Fig. 4B). While *cC*OMVs served as negative control. *Kp* and *cC*OMVs were added to HULEC-5a cells at MOV_1000_ for 180 min with or without PB pretreatment for 1 h. Combination of *Kp*OMVs with PB blocked degradation of IRAK-1 and phosphorylation of p38. In contrast to *Kp*OMVs, *cC*OMVs lacked the capacity to induce degradation of IRAK-1 along with phosphorylation of p38 (Fig. 4B).

Additionally, nuclear translocation of p65 and p38 were investigated as mean of NF-κB and p38 signaling activation and their nuclear translocation both occurred 30 min after *Kp*OMV addition (Fig. 4C). Interestingly, PB pretreatment of *Kp*OMVs reduced p65 and p38 translocation (Fig. 4D). Altogether, these results indicate that OMVs induce LPS-mediated TLR4 signaling and associated downstream activation via the MyD88/IRAK-1 axis that further results in activation of NF-κB and p38 signaling to block RNase1 in human ECs.

### OMVs repress endothelial RNase1 mRNA via p38 signaling

**Fig. 5:**
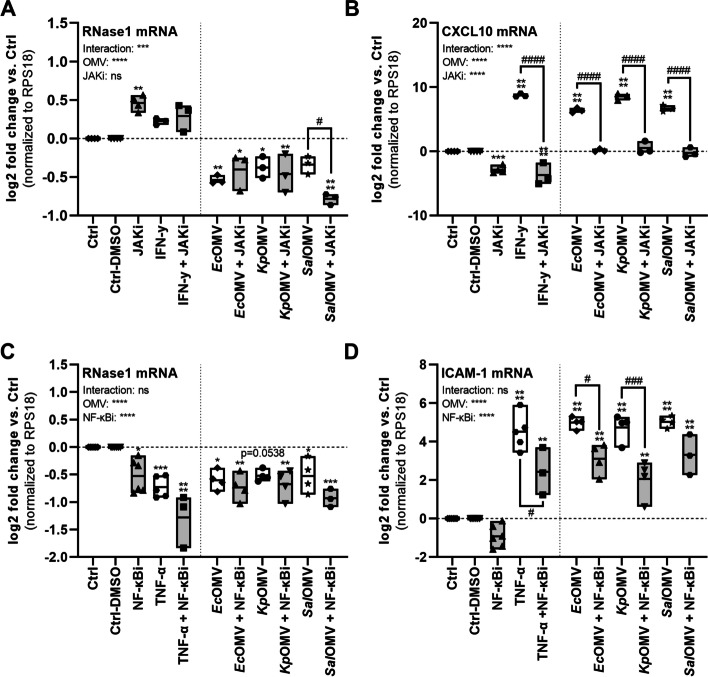
OMV-mediated RNase1 mRNA repression is independent of JAK/STAT and NF-κB signaling. HULEC-5a were pretreated for 1 h with 5 µM JAKi (Ruxolitinib) or NF-κBi (BAY-11–7082) followed by 16 h stimulation with SEC-purified OMVs from *Ec*, *Kp* and *Sal* (MOV_1000_) as well as IFN-γ (250 ng/ml) or TNF-α (10 ng/ml) as indicated, left untreated as control (Ctrl) or treated with DMSO as solvent control (Ctrl-DMSO). mRNA expression of RNase1 (**A** and **C**), **B**) CXCL10 or **D**) ICAM-1 was determined by qPCR, normalized to RPS18 and the respective Ctrl. *n* = 3–6, line at mean, Two-way ANOVA with Tukey‘s multiple comparison test. * compared to corresponding Ctrl, # as indicated. *p < 0.05, **p < 0.01, ***p < 0.001, ****p < 0.0001. ^#^*p* < 0.05, ^##^*p* < 0.01, ^####^*p* < 0.0001

**Fig. 6:**
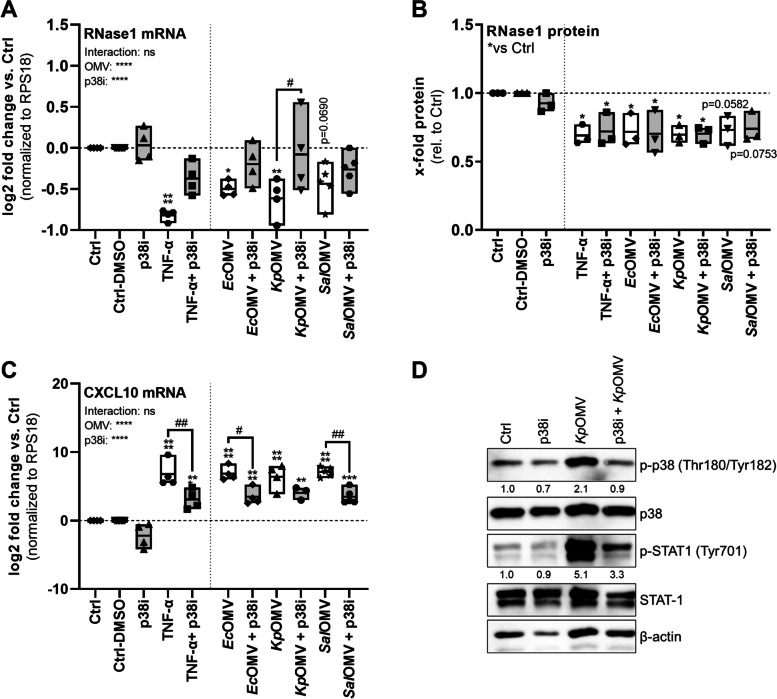
OMV-mediated RNase1 mRNA repression depends on p38 signaling. HULEC-5a were pretreated for 1 h with 10 µM p38i (SB202190) followed by 16 h stimulation with SEC-purified OMVs from *Ec*, *Kp* and *Sal* (MOV_1000_) as well as TNF-α (10 ng/ml), treated with DMSO as solvent control (Ctrl-DMSO) or left untreated as control (Ctrl). mRNA expression of **A**) RNase1 and **C**) CXCL10 was determined by qPCR, normalized to RPS18 and the respective Ctrl. *n* = 3–5, line at mean, Two-way ANOVA with Tukey‘s multiple comparison test. **B)** RNase1 protein release from 16 h stimulated HULEC-5a was measured by ELISA, depicted as x-fold protein relative to Ctrl. **D)** HULEC-5a were pretreated with p38i (10 µM for 1 h) followed by 180 min *Kp*OMV stimulation (MOV_1000_). Expression and phosphorylation of p38 and STAT1 were determined by Western Blot. β-actin served as a loading control. One representative result is shown. **A, C)** *n* = 4, line at mean; Two-way ANOVA with Tukey‘s multiple comparison test. * compared to corresponding Ctrl, # as indicated. *p < 0.05, **p < 0.01, ***p < 0.001, ****p < 0.0001. ^#^*p* < 0.05, ^##^*p* < 0.01. **B)** *n* = 3, line at mean; One-way ANOVA with Tukey ‘s multiple comparison test. *p < 0.05

To further identify the responsible downstream signaling pathway for OMV-mediated RNase1 repression, we performed OMV-stimulation experiments combined with signaling molecule inhibitors targeting the type I interferon- or IL-6-mediated JAK/STAT pathway (JAKi: JAK1 inhibitor; Ruxolitinib; 5 µM), the MyD88/IRAK-1/NF-κB pathway (NF-κBi: NF-κB inhibitor; BAY11-7082; 5 µM) and the MyD88/IRAK-1/p38 pathway (p38i: p38 inhibitor; SB202190; 10 µM). Therefore, HULEC-5a were pretreated for 1 h with JAKi, NF-κBi (Fig. 5) or p38i (Fig. 6; grey bars) prior to 16 h OMV (MOV_1000_), IFN-γ (250 ng/ml) or TNF-α (10 ng/ml) stimulation (white bars) as indicated. Untreated and DMSO treated cells served as controls (Ctrl, Ctrl-DMSO). Investigation of JAK/STAT signaling using JAKi showed a slight upregulation of RNase1 mRNA by the inhibitor itself compared to the respective controls. Additionally, IFN-γ as an initiator of JAK/STAT signaling only slightly affected RNase1 mRNA levels without reaching significance, that was not affected by JAKi treatment (Fig. 5A). In line with our previous data for STAT1 phosphorylation (Fig. 4A), RNase1 mRNA expression was repressed upon *Ec*OMV, *Kp*OMV and *Sal*OMV stimulation independent of JAKi treatment (Fig. 5A). In addition, mRNA expression of the JAK/STAT-dependent mRNA CXCL10 [49] was significantly increased upon IFN-γ and OMV treatment that was blocked to basal level by JAKi pretreatment, indicating successful pathway inhibition by the applied inhibitor (Fig. 5B). As JAK/STAT signaling seems not to be responsible for OMV-mediated RNase1 regulation, we further investigated the MyD88/IRAK-1/NF-κB pathway using NF-κBi (Fig. 5C-D). Interestingly, the inhibitor itself slightly repressed RNase1 mRNA expression and could not recover TNF-α- or OMV-mediated RNase1 repression on mRNA level. On the contrary, NF-кBi further intensified the repressive effect of TNF-α and OMVs on RNase1 expression (Fig. 5C). Functionality of the inhibitor was demonstrated by mRNA expression of NF-κB-dependent ICAM-1 [50, 51]. Here, TNF-α or OMV induced ICAM-1 mRNA expression was successfully reduced upon NF-κBi treatment compared to samples without inhibitor pretreatment (Fig. 5D).

As a third potential pathway regulating OMV-mediated RNase1 repression, the MyD88/IRAK-1/p38 axis was analyzed using p38i (Fig. 6). Similar to previous data obtained with PB-pretreated OMVs, p38i preincubation (grey bars) of HULEC-5a markedly recovered RNase1 mRNA expression after treatment with TNF-α, *Ec*OMV, *Kp*OMV or *Sal*OMV (white bars) to almost basal levels compared to p38i alone or the respective controls. Furthermore, this effect even reached significance in the case of *Kp*OMVs (Fig. 6A). Interestingly, RNase1 protein secretion was significantly repressed by TNF-α and OMV exposure independent of p38i treatment, which did not rescue the protein release (Fig. 6B). In addition, TNF-α, *Ec*OMV, *Kp*OMV or *Sal*OMV induced CXCL10 mRNA expression was significantly reduced by p38i, verifying a functional inhibition (Fig. 6C) [49, 52], which is further promoted by successful blocking of p38 phosphorylation upon *Kp*OMV treatment and reduced phosphorylation of STAT1 (Fig. 6D). Additionally, stimulation of HULEC-5a with all aforementioned stimuli and inhibitors was not cytotoxic to the cells (Fig. S3E-G).

Altogether our data provides evidence that OMV-mediated RNase1 mRNA repression is regulated via the TLR4/MyD88/IRAK-1/p38 cascade in human lung endothelial cells.

## Discussion

Sepsis is a life-threatening condition caused by a deregulated immune response to infection that leads to organ dysfunction and accounts for almost 20% of all global mortalities [1, 53]. In this context, vascular dysfunction is a key feature of sepsis, which can lead to bleeding, multi-organ failure and death [8, 9, 54]. Besides the classical inflammatory mediators like cytokines or thrombotic factors, EVs were found to be key players in intercellular communication during infection [11]. Bacteria-derived EVs can be secreted into the blood stream during systemic infection, but are also able to reach the blood prior to the bacteria and disseminate further and faster [14, 55]. Moreover, they are capable of causing-sepsis like systemic inflammation [56] and are associated with complications of sepsis such as disseminated intravascular coagulation and cardiac malfunction [18, 57, 58].

To understand how bEVs influence sepsis progression and endothelial dysfunction, we investigated the impact of bEVs from the sepsis-associated pathogens *Escherichia coli*, *Klebsiella pneumoniae*, *Salmonella enterica* serovar Typhimurium and *Streptococcus pneumoniae* on RNase1, a vessel protective factor, in human lung ECs. Gram-negative OMVs from *Ec*, *Kp* and *Sal* significantly reduced RNase1 expression compared to the gram-positive MVs from *Sp*. Furthermore, we demonstrated that the OMV-mediated RNase1 repression is regulated via LPS-induced activation of the TLR4/MyD88/IRAK-1 axis, with p38 playing a crucial role in inflamed human lung ECs.

RNase1 is a vessel-protective factor that counteracts the effects of eRNA, whose downregulation is linked to various vascular pathologies, like thrombosis, myocardial infarction, atherosclerosis and stroke [30, 41]. Although insufficiently studied in bacterial infections and sepsis, RNase1 administration has been shown to block the eRNA-mediated mechanism of alveolar epithelial cell infection by *Streptococcus pneumoniae* [59]. Additionally, RNase1 levels are elevated in serum in the early disease stage of sepsis and can act as a prognostic factor for the development of multi-organ failure [43]. However, sepsis progression leads to an increase in serum levels of RNase1 antagonists, eRNA and RNase-Inhibitor, potentially repressing RNase1. Besides that, studies in mice suggest that RNase1 administration can prevent sepsis-associated tissue and organ damage [42], highlighting its potential as a therapeutic intervention. These findings, combined with the profound influence of RNase1 repression in thrombotic diseases, suggest that the RNase1-eRNA system plays a crucial role in infectious and systemic diseases like sepsis, potentially promoting disease progression and a fatal outcome.

Future investigation into RNase1 in the context of (pneumogenic) sepsis is crucial, as pneumonia is the leading cause of sepsis. To this end, we investigated the impact of bEVs from sepsis-associated pathogens on RNase1 regulation in human lung microvascular ECs (HULEC-5a) as a model system. ECs in the pulmonary microvasculature play a critical role in gas exchange [60, 61] and also act as first line of defense by initiating the immune response against invading pathogens [62].

We found that only OMVs from the gram-negative bacteria *Ec*, *Kp* and *Sal* activated the endothelium and repressed RNase1, while gram-positive MVs from *Sp* could not. The difference may be due to surface-exposed bacterial toxins, with lipoprotein being the major toxin exposed on *Sp*MVs and LPS being present on OMVs [63, 64]. Previous studies demonstrated that *Sp*MVs activated proinflammatory signaling cascades in primary human macrophages via exposed lipoprotein and TLR2 [44, 63, 65]. HULEC-5a may be insensitive to *Sp*MVs due to low TLR2 expression, whereas TLR4 is expressed and cells were more responsive to OMV-associated LPS from *Ec*, *Kp* and *Sal*. This is in line with the literature, showing low expression of TLR2 and high expression of TLR4 in dermal microvascular ECs [66].

As consequence, OMVs from *Ec*, *Kp* and *Sal* robustly activated the endothelium, that is consistent with data from macrophages [44, 67], as indicated by increased expression of the key inflammatory cytokines CXCL8 and CXCL10 and the cell adhesion molecule ICAM-1 [68, 69] and repression of RNase1 mRNA and protein. This effect might be attributed to the OMV-exposed LPS, as confirmed by treatment with Polymyxin B (PB), an LPS neutralizing peptide antibiotic [46], that could block the OMV-mediated RNase1 repression and EC activation. In comparison, OMVs from *cC* carrying a non-functional LPS did not influence RNase1 or the proinflammatory EC activation. These data are in line with literature that demonstrates a potent function of PB in blocking OMV-exposed LPS-mediated inflammation [70, 71].

The RNase1 recovering effect of PB in HULEC-5a indicated an LPS/TLR4-dependent mechanism responsible for inducing RNase1 repression. Investigation of the underlying intracellular signaling induced by OMV stimulation of human lung ECs revealed activation of both the MyD88/IRAK-1 and TRIF axes [23–25, 68]. These findings align with literature investigating OMV-mediated signal transduction, where *Legionella pneumophila* OMVs were shown to proinflammatory activate macrophages via TLR2, IRAK-1 and NF-кB [72], while OMVs from *Porphyromonas gingivalis* mediate endothelial nitric oxide synthase suppression in human ECs via ERK1/2 and p38 signaling [73]. Interestingly, PB treatment of OMVs only blocked the activation of the MyD88/IRAK-1 axis as indicated by reduced IRAK-1 degradation, p38 phosphorylation and translocation and p65 translocation. These results point towards a MyD88/IRAK-1-dependent signaling cascade for RNase1 repression via NF-кB and p38. Similar regulations of LPS-induced TLR4 signaling and PB were obtained by Cheng et al., who demonstrated an impact of PB on LPS-induced endotoxemia in mice via the TLR4/MyD88 axis [74]. Signaling pathway inhibitors targeting specific downstream molecules of TLR4 were used for verification. The JAK1 inhibitor Ruxolitinib was used to investigate the TLR4/IRF axis that provokes activation of IFN signaling via JAK1/STAT1 [23–25]. However, Ruxolitinib treatment of HULEC-5a prior to OMV stimulation did not prevent RNase1 repression and Ruxolitinib treatment alone increased RNase1 expression. Little is known about the underlying molecular pathways of RNase1 regulation in ECs, but referred data primarily focused on p38 and histone deacetylase 2 (HDAC2)-mediated mechanisms [35, 36]. Besides the possibility of off-target effects of Ruxolitinib, JAK1 can also be associated to IL-6 signaling [75]. Unpublished data by our group suggest that HULEC-5a produce IL-6 in response to OMV stimulation, and IL-6/IL-6R treatment represses RNase1. Thus, it might be possible that JAK1 can be associated to RNase1 signaling via an autocrine feedback loop in which OMVs trigger the release of IL-6 via TLR4/NF-кB/p38 signaling [68, 76, 77]. This in turn can reduce RNase1 expression via JAK1 as well as self-perpetuates IL-6 to promote persistent inflammation. As IL-6 is also known as a key inflammatory mediator during cytokine storm in sepsis [2], further investigation of a possible impact of Ruxolitinib, JAK1 and IL-6 in RNase1-associated EC dysfunction might be of future interest. Thereby, Ruxolitinib could be suitable to prevent sepsis-associated IL-6 release to impede EC dysfunction via promoting RNase1 expression.

As the downstream signaling of TLR4/MyD88/IRAK-1 can either be mediated via NF-κB or p38, we further investigated the impact of these on OMV-mediated RNase1 repression using BAY11-7082 as NF-κB inhibitor and SB202190 as p38 inhibitor. BAY11-7082 itself slightly downregulated RNase1 and elevated the repressive effect of TNF-α and OMVs. However, previous studies by Gansler et al. and our group did not observe any impact of NF-κB inhibition on RNase1 mRNA expression in primary human umbilical vein ECs [34, 35]. Furthermore, JNK signaling was found to be important for physiological RNase1 expression, as JNK inhibition repressed RNase1 [35]. These findings suggest that RNase1 regulation in ECs may be organ-specific and vary depending on the physiological demands of the vascular bed [78–81]. ECs from different organs can activate  organ-specific pathways and express organ-specific transporters and surface markers [60], and even within a specific vascular bed, there can be differences in EC function [82]. This heterogeneity may explain differences in reactivity of the microvascular lung endothelium used in this study compared to ECs from the large umbilical vein used in previous studies addressing RNase1 regulations.

Compared to these findings, blocking the p38 signaling cascade with SB202190 recovered RNase1 mRNA, indicating that OMV-mediated TLR4 activation signals via MAPK p38 to repress RNase1 expression which is in line with previous studies on TNF-α-mediated RNase1 mRNA repression [35]. TNF-α is a major regulator of RNase1 [39, 41, 83, 84] and can repress it through activation of an HDAC2-containing NuRD repressor complex, which deacetylates the *RNASE1* promoter region and prevents RNase1 transcription [36]. This pathway is mediated via p38 signaling as its inhibition impedes repressor complex recruitment and *RNASE1* chromatin modulation upon TNF-α stimulation [35, 36]. Therefore, p38 MAPK negatively regulates gene expression and is associated with known RNase1 repressive stimuli like TNF-α or IL-1β as well as LPS [35, 85–87]. Although RNase1 mRNA expression was recovered by p38 inhibition, its protein release was still affected by OMVs as well as TNF-α treatment. As p38 is known as a major regulator of intracellular signaling, inhibition of this pathway can also influence a variety of cellular factors that might trigger RNase1 repression [85, 86, 88, 89]. Based on our data we conclude that an unknown factor that acts downstream of TLR4 affects RNase1 protein release, as PB treatment efficiently recovered RNase1 protein upon OMV stimulation. However, further investigation needs to be done to maintain RNase1 integrity in the inflamed endothelium. The presented results provide strong evidence for a p38-mediated RNase1 mRNA regulation upon OMV treatment and identify p38 as a key repressor of RNase1, consistent with our previously published data on TNF-α mediated RNase1 regulation [35, 36].

Furthermore, our study suggests potential therapeutic strategies for sepsis-associated vascular dysfunction by restoring RNase1 function and vascular homeostasis. While p38 inhibitors have shown promise in clinical trials [90–93] by decreasing serum levels of proinflammatory cytokines [94], our data indicates that p38 inhibition alone might not be sufficient to restore RNase1 function and vascular integrity. Thus, a combination of p38 inhibitors with other complementary therapeutic options should be considered. One particularly interesting option is PB treatment, as it represents an approved drug and has already been investigated in several clinical trials for sepsis intervention, where it improved various clinical outcomes, such as mean arterial pressure, ventilator-free days, and mortality [95–99]. PB treatment fully recovered RNase1 mRNA and protein and has been shown to reduce the proapoptotic function of plasma of septic patients in sepsis-induced acute renal failure [100]. Additionally, new innovations such as PB-releasing nanoparticles have been developed to target bacterial accumulation and vascular inflammation [101] and thereby could be a valuable tool for sepsis intervention through RNase1 recovery in human ECs.

## Conclusions

In summary, we observed that EVs released by different gram-negative, (pneumogenic) sepsis-associated bacteria repress RNase1, a key modulator of vascular integrity, in human lung microvascular ECs. This effect is mediated via signaling through TLR4/MyD88/IRAK-1 and p38 and can be prevented by LPS neutralization via PB and in part p38 inhibition. Thereby, our data provides new insights in the regulation of sepsis-induced vascular dysfunction and offers novel treatment options to prevent sepsis-associated RNase1 repression and consecutive vascular breakdown.

## Supplementary Information

**Additional file 1: Figure S1.** OMV-mediated intracellular signaling cascades. **Figure S2.** Isolation procedure and characterization of bEVs. **Figure S3.** Impact of OMVs and inhibitor treatments on HULEC-5a. **Figure S4.** SpMVs do not activate human lung ECs. **Figure S5.** Basal TLR2 and TLR4 mRNA expression in human lung ECs.

## Abbreviations

bEVs: Bacterial extracellular vesicles
*cC*: *Clear Coli*
CXCL8: C-X-C motif chemokine ligand 8
CXCL10: C-X-C motif chemokine ligand 10
DMSO: Dimethyl sulfoxide
*Ec*: *Escherichia coli*
eRNA: Extracellular RNA
EVs: Extracellular vesicles
HDAC2: Histone deacetylase 2
ICAM-1: Intercellular adhesion molecule 1
IFN: Interferon
IFNAR: Interferon alpha/beta receptor
JAKi: Janus activated kinase inhibitor (Ruxolitinib)
*Kp*: *Klebsiella pneumoniae*
LDH: Lactate dehydrogenase
LPS: Lipopolysaccharide
MAPK: Mitogen activated protein kinase
MVs: Membrane vesicles
NF-кBi: Nuclear factor kappa B inhibitor (BAY11-7081)
OMVs: Outer membrane vesicles
p38i: P38 inhibitor (SB202190)
PB: Polymyxin B
qPCR: Quantitative reverse transcription PCR
RNase1: Ribonuclease 1
*Sal*: *Salmonella enterica* serovar Typhimurium
*Sp*: *Streptococcus pneumoniae*
TLR: Toll-like receptor
TNF-α: Tumor necrosis factor alpha
UF-SEC: Ultrafiltration and size exclusion chromatography
