# Supplementary material for: Bacterial extracellular vesicles repress the vascular protective factor RNase1 in human lung endothelial cells
Source: Cell Commun Signal. 2023 May 15;21:111. doi: 10.1186/s12964-023-01131-2 (PMC10184351; doi:10.1186/s12964-023-01131-2)
Supplement: Supplementary file 2 — Additional file 1: Figure S1. OMV-mediated intracellular signaling cascades. Figure S2. Isolation procedure and characterization of bEVs. Figure S3. Impact of OMVs and inhibitor treatments on HULEC-5a. Figure S4. SpMVs do not activate human lung ECs. Figure S5. Basal TLR2 and TLR4 mRNA expression in human lung ECs. [file 12964_2023_1131_MOESM1_ESM.docx]

**Supplementary data**

**Manuscript title: Bacterial extracellular vesicles repress the vascular protective factor RNase1 in human lung endothelial cells**

Running title: RNase1 repression by bacterial vesicles

**Figure S1:**

**
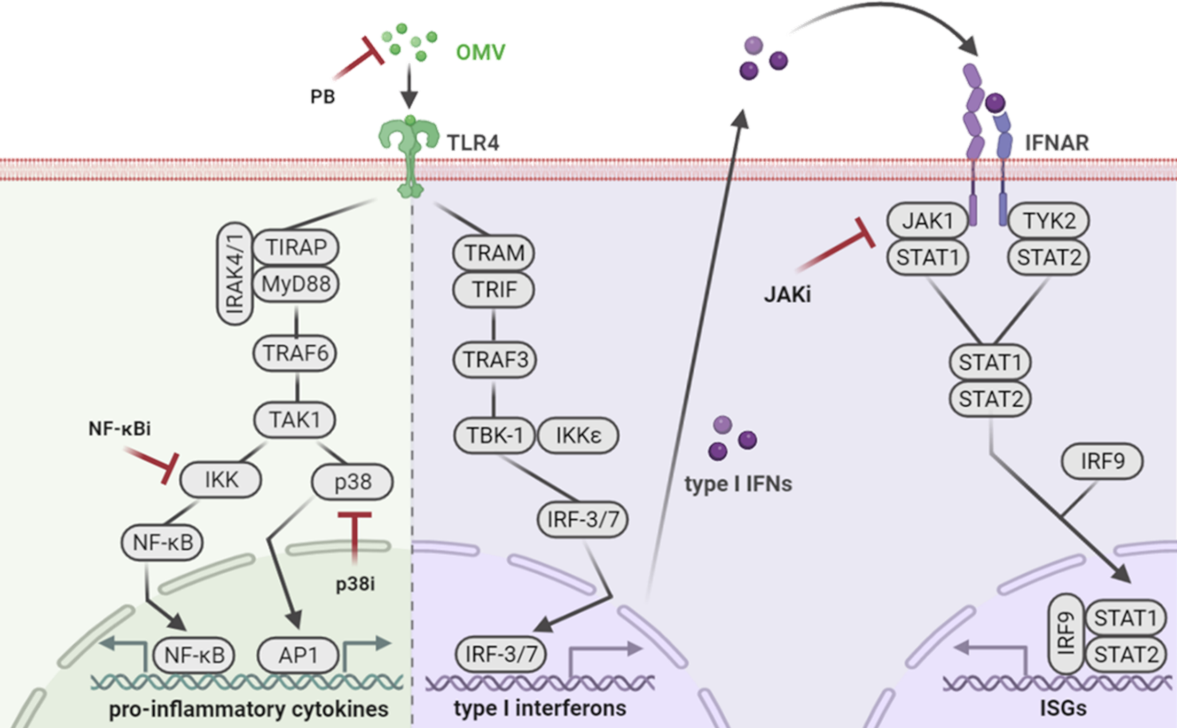
**

**Figure S1: OMV-mediated intracellular signaling cascades.** Schematic overview of potential OMV-induced signaling cascades via TLR4. TLR4 activation induces intracellular signaling via either the MyD88/IRAK-1 or the TRIF axis, thereby activating IRAK-1 degradation, NF-κB translocation and p38 phosphorylation and translocation to the nucleus to activate associated gene expression. Additionally, TLR4 can induce TRIF-dependent signaling that promotes IRF3/7-mediated type I IFN production and subsequent activation of type I interferon receptor (IFNAR) and the JAK/STAT pathway. Specific signaling pathway inhibitors used in this study are indicated in red (PB: Polymyxin B, JAKi: Ruxolitinib, NF-κBi: BAY11-7081, p38i: SB202190). Created with Biorender.com

**Figure S2:**


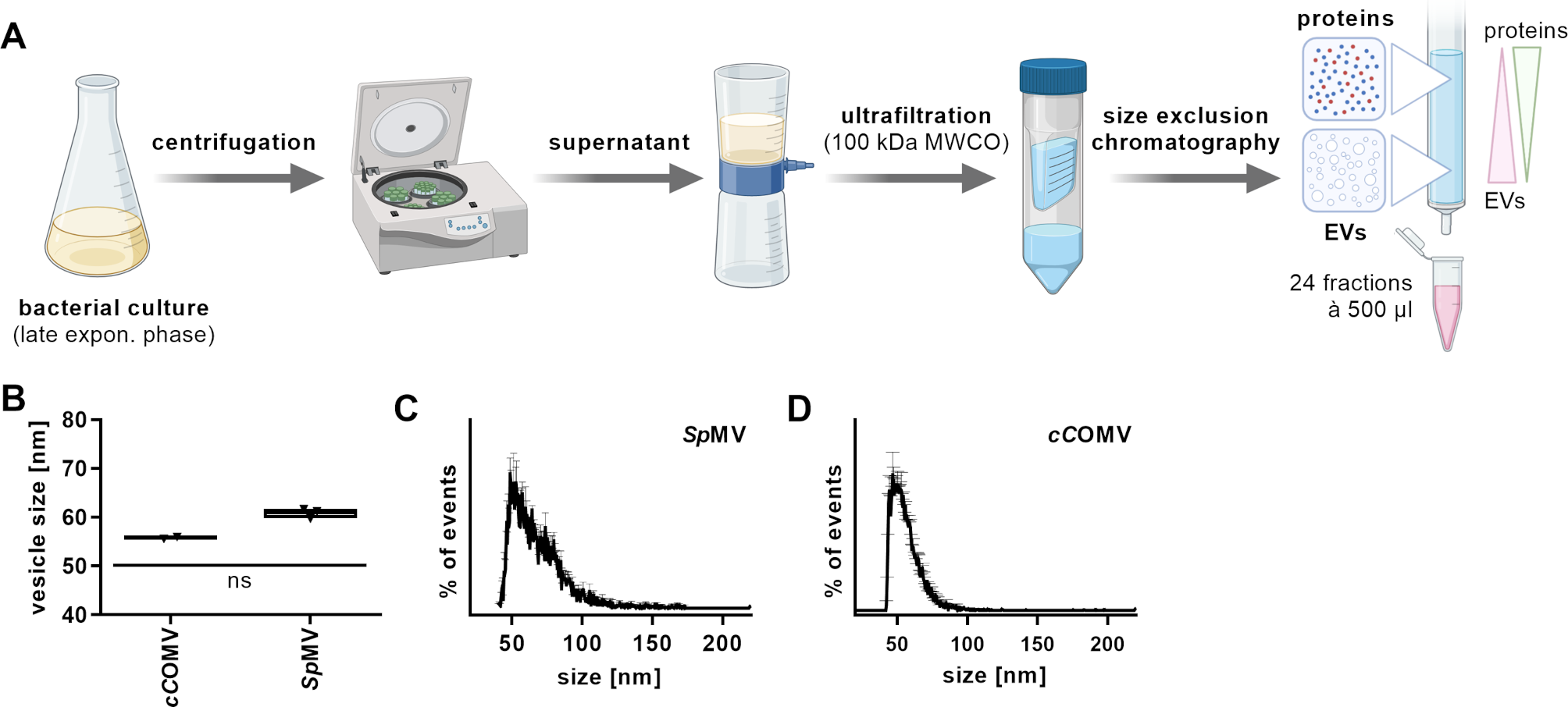


**Figure S2: Isolation procedure and characterization of bEVs.** A) Schematic overview of the OMV/MV isolation procedure via centrifugation, ultrafiltration and size exclusion chromatography. Created with Biorender.com. B) Mean size of SEC-purified vesicles from *Clear coli* (*cC*OMV) and *Streptococcus pneumoniae* (*Sp*MV) and their size distribution profile (C-D) was determined by nano Flow Cytometry (nanoFCM). n=2-3, line at mean. B) One-way ANOVA with Tukey‘s multiple comparison test; ns: not significant.

**Figure S3:**


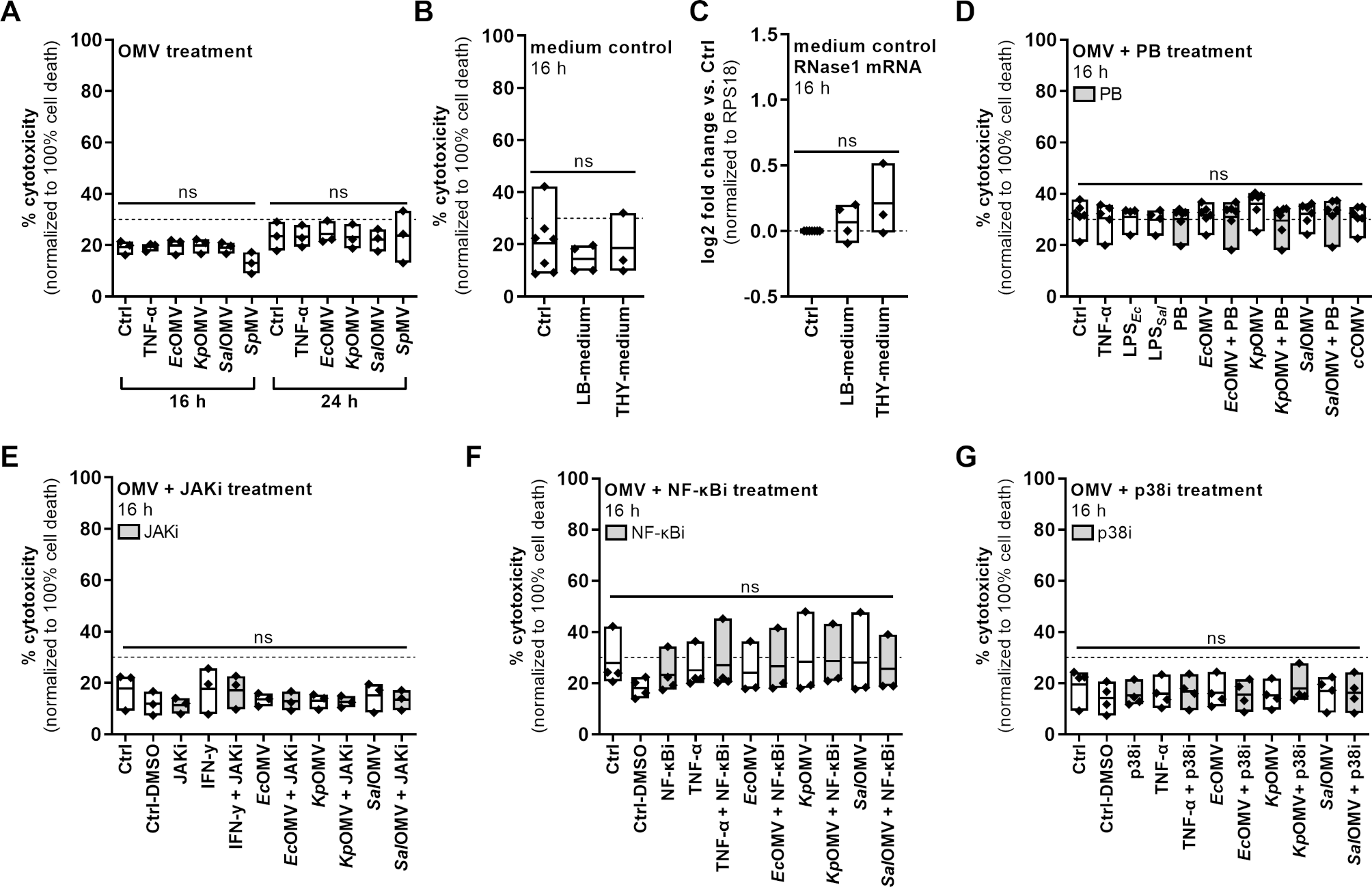


**Figure S3: Impact of OMVs and inhibitor treatments on HULEC-5a.** HULEC-5a were treated for 16 h (A-G) and 24 h (A) with OMVs/MVs (MOV_1000_), LPS from *Sal* or *Ec* (LPS*_Sal_* or LPS*_Ec_*: 100 ng/ml), TNF-α (10 ng/ml), IFN-γ (250 ng/ml), LB- or THY-medium as medium control in combination with or without 1 h inhibitor pretreatment. A-C) without inhibitor, D) PB (Polymyxin B): 20 µg/ml, E) JAKi (Ruxolitinib): 5 µM, F) NF-κBi (BAY11-7082): 5 µM or G) p38i (SB202190): 10 µM. A-B, D-G) Cytotoxicity (%) was measured by LDH-Assay from supernatants compared to a total lysis, defined as 100 % cell death. Threshold set to 30% cell death (scattered line). C) mRNA expression of RNase1 was analyzed by qPCR, normalized to RPS18 and untreated cells (Ctrl). n=3-6. line at mean. One-way ANOVA with Dunnett‘s multiple comparison test compared to Ctrl. ns: not significant.

**Figure S4:**


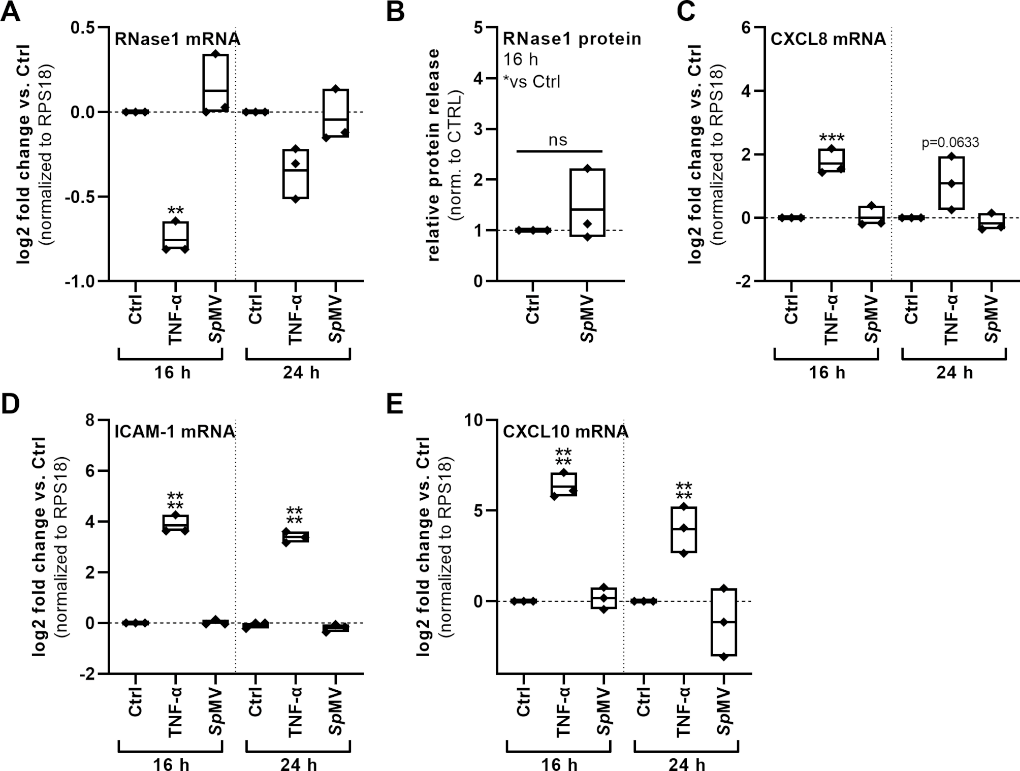


**Figure S4: *Sp*MVs do not activate human lung ECs.** HULEC-5a were stimulated for 16 h and 24 h with SEC-purified MVs (MOV_1000_) from *Sp* or TNF-α (10 ng/ml) or left untreated as control (Ctrl). mRNA expression of A) RNase1 C) CXCL8, D) ICAM-1 and E) CXCL10 was determined by qPCR, normalized to RPS18 and untreated cells (Ctrl). B) RNase1 protein release in supernatants of 16 h stimulated HULEC-5a was measured by ELISA, depicted as x-fold protein relative to control (Ctrl). n=3, line at mean, A, C-E) One-way ANOVA with Dunnett‘s multiple comparison test compared to respective Ctrl. **p<0.01, ***p<0.001, ****p<0.0001. B) unpaired t-test. ns: not significant.

**Figure S5:**


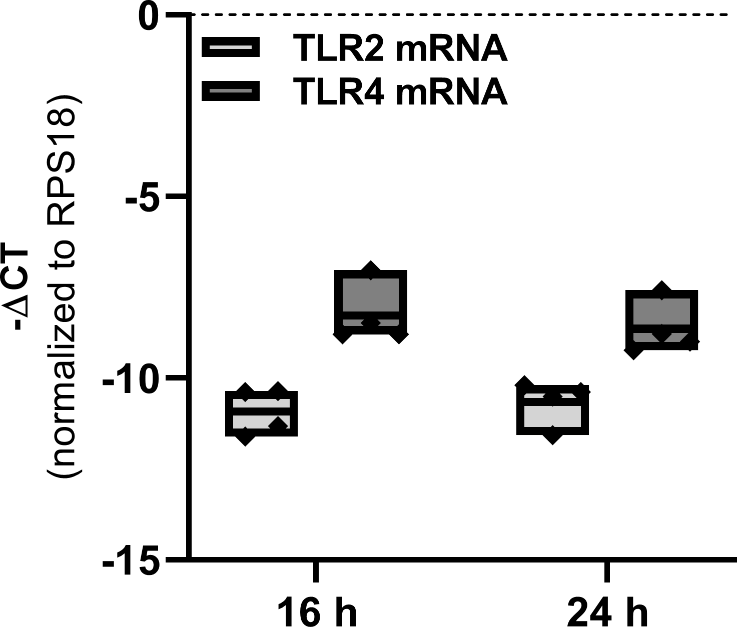


**Figure S5: Basal TLR2 and TLR4 mRNA expression in human lung ECs.** Untreated HULEC-5a were used for determination of basal mRNA expression of TLR2 (light grey) and TLR4 (dark grey). qPCR results were normalized to RPS18 and depicted as -∆CT. n=4.
